# Simultaneous systematic analysis approach changing the paradigm

**DOI:** 10.1186/1878-5085-5-S1-A154

**Published:** 2014-02-11

**Authors:** Mira Marcus-Kalish

**Affiliations:** 1 Tel Aviv University, Israel

## 

**Figure 1:**
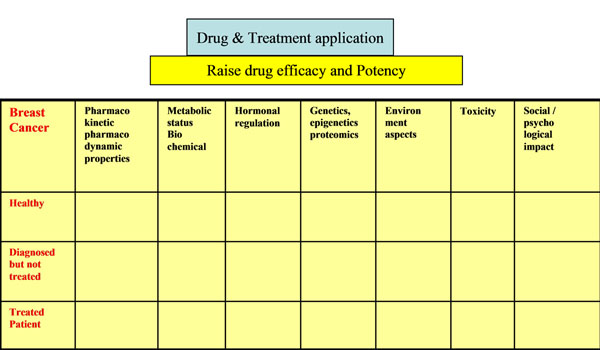
**The Matrix Approach** applied to a specific disease. Simultaneous Systematic Analysis and joint efforts among all research groups and professionals.

The surge of the new interdisciplinary centers, the new titles and slogans, these days, are all a genuine attempt to lead a responsible change of the research and development as well as the treatment paradigm towards better healthcare in particular, and well being to ALL, in general. The goal is to change the paradigm in health care - from delayed interventional medicine to predictive personally tailored medicine, from reactive to preventive medicine and from disease to wellness, facing the major needs of society. Based on the remarkable achievements in the past decade, the CKTS (Converging Knowledge, Technology and Society) approach was presented by the NSF (Dec. 2012) reflecting the series of workshops held around the world. The “Holistic approaches to wellness and human development", is one focus, among other wide range of recommendations in the broad area of science. That is fully aligned with the practice of medicine that is moving from a passive coincidence model to an active convergence model, including micro and macro environmental effects. At the center of this transformation is the intersection of physiology, molecular profiling and information technology, combined with a number of converging technologies that affected the human cognition, communication and quality of life. The common denominators were the availability and compatibility of technologies, functional imaging including brain imaging, enabling of big data analysis, etc. The practical goal is to provide inspired translational research from basic science to preventive, predictive and personalized medicine. As a practical enabling system, a Simultaneous Systematic Analysis (SSA) approach is suggested, to provide the broadest possible insight into the individual comprehensive functioning in the surroundings - as one refined complex system- over time and modes of actions. The targeted platform envisioned will enable and encourage an open, innovative systematic effort, sharing all findings and combining all acquired knowledge, technologies and expertise, focused on a specific phenomenon or disease. The approach suggests an operational mode for multilevel, multisource research that would reduce barriers, encourage creativity and innovative research, while enabling responsible and safe sharing of all findings. It can be envisioned as a matrix scheme, applied, for example, to drug efficacy, in which the rows represent the various levels of the patient (healthy, diagnosed but not treated, treated, etc) and the columns represents the various research and professionals working groups, each focusing on a specific aspect of the micro- and macro-environment (see figure 1). The goal is to involve all stakeholders (industry, governmental- policy, regulatory, patients, etc) and the professionals at all R&D levels, in an ongoing structural and coherent discussions. The CHEST (Converging Humanities, Education, Science and Technology) methodology would be utilized to provide the platform for open discussion, while bridging the gaps and reducing any formal or conceptual barriers between all partners. The idea is to bring on board the ethics professionals, for example, even at the basic science level, discuss all mental and physical aspects, embed the "caution" and "cognition" points of view etc - all which will ensure innovative thinking as well as reliable, balanced and profitable solutions to All. These creative operational schemes yielding the essential mutual fertility, could shorten the lead time to mechanism understanding and thus are essential in enabling personalized and preventive medicine, providing best fitting treatments and products. Applying this approach to drug and treatment applications, as presented in the matrix, may have a broad economical and sociological impact yielding demographic changes and thus political issues.

**Figure 2:**
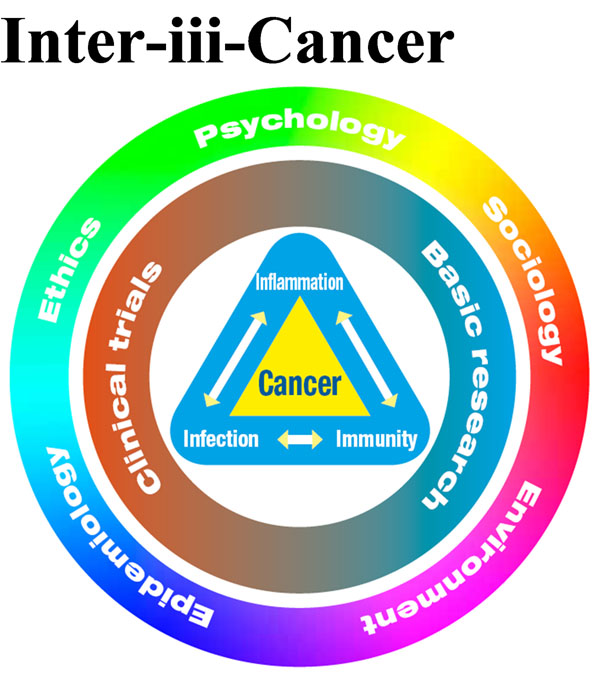
The Center for Interdisciplinary Research on Infection-Immunity-Inflammation Driven Cancer Diseases

The iii-3 Cancer Center - (Infection-Immunity-Inflammation Driven Cancer Diseases), at Tel Aviv University, is another feasibility study, as an example, to the SSA approach (see figure 2). The goal is to combine the study of all micro features (physiology, genetics, biomarkers, etc) at all levels with the macro features such as environmental studies, ethics, psychology, community etc, in order to provide inspired translational research from basic science to personalized medicine at the cancer Infection- Immunity- Inflammation focused group. The Infection - Immunity - Inflammation triangle is at the heart of this approach, as a core factor in cancer development and treatment, including the interplay with the surrounding factors. Through changing the paradigm and combining all relevant research areas and know-how in the lab and clinic, iii-Cancer will enable to advance towards innovative reliable preventive, predictive and personalized cancer treatment.

In order to apply the SSA approach, as true for other initiatives, further formal and managerial steps have to be taken by regulatory and policy makers agencies, as a pre-requisite for applying such joint open flows working schemes:

- ensuring intellectual property, simplifying and shortening the processes.

- shortening the peer review process (as was mentioned by Leshner in AAAS 2011)

- shortening the time-lag for patent administration - the regulatory scheme and procedures.

- education paradigm change at all formal levels from childhood to graduate studies.

- academic institute's structure and culture adaption at all levels: teaching and collaborative research paradigm including adjusting the personal evaluation and promotion schemes.

